# Corn Bran IDF and Polyphenol-Reduced IDF Alleviate Low-Dose 3-MCPD-Induced Toxicity by Modulating Gut Microbiota and Intestinal Barrier

**DOI:** 10.3390/foods14244253

**Published:** 2025-12-10

**Authors:** Zhiqiang Song, Huanxiao Jiang, Huiying Dai, Liying Bo, Chunli Song, Xiaolan Liu, Jian Ren

**Affiliations:** Engineering Research Center of Plant Food Processing Technology, Ministry of Education, Qiqihar University, Qiqihar 161006, China02982@qqhru.edu.cn (H.J.); a11613230@163.com (H.D.); boliying1746@126.com (L.B.); songchunli@qqhru.edu.cn (C.S.); liuxiaolan001@126.com (X.L.)

**Keywords:** insoluble dietary fiber, 3-MCPD, motor impairment, intestinal barrier, gut microbiota

## Abstract

The polyphenols in insoluble dietary fiber (IDF) alleviate the toxicity of 3-chloro-1,2-propanediol (3-MCPD); however, the effect of polyphenol-reduced IDF on 3-MCPD toxicity remains unclear. This study compared the effects of IDF and IDF modified with alkaline hydrogen peroxide (IDF-AHP) on alleviating the toxicity of low-dose 3-MCPD in mice. The results indicate that both IDF and IDF-AHP retained their polysaccharide structures; however, the polyphenol content and antioxidant activity of IDF-AHP were significantly reduced. Both fibers alleviated 3-MCPD-induced motor impairments. Histological studies revealed that both dietary fibers reduced inflammatory cell infiltration in the colon, decreased serum diamine oxidase (DAO) levels, and enhanced intestinal barrier function. 16S rRNA sequencing results indicated that the two dietary fibers did not affect the abundance or uniformity of the microbiota within individual samples but did cause differences in the microbial composition between samples. *Alistipes* and *Bacteroides* (IDF + 3-MCPD group vs. 3-MCPD group) and *Mucispirillum* (IDF-AHP + 3-MCPD group vs. 3-MCPD group) may mediate the effects of IDF and IDF-AHP in alleviating motor impairment induced by low-dose 3-MCPD. These findings suggest that the attenuation of low-dose 3-MCPD toxicity by IDF and polyphenol-reduced IDF may be related to the modulation of gut barrier function and the abundance of gut microbiota. They have potential as food additives and hold promise for further development as functional foods.

## 1. Introduction

3-Monochloropropane-1,2-diol (3-MCPD) is a food contaminant classified among the class of chloropropanols, raising concerns for both the food industry and regulatory authorities [1]. 3-MCPD exhibits multiple toxic effects, including reproductive toxicity, nephrotoxicity, neurotoxicity, immunotoxicity, carcinogenicity, genotoxicity, and metabolic toxicity, all of which pose significant risks to human health [2]. It is widely acknowledged that this contaminant cannot currently be completely eliminated from food products or food processing methods [3]. Chronic consumption of foods containing 3-MCPD may lead to adverse suboptimal health effects in humans. Incorporating dietary supplements may be an effective strategy to enhance the nutritional value of foods while mitigating or eliminating the toxicity associated with 3-MCPD. Various supplements, including tea polyphenols [4], cyanidin-3-O-glucoside [5], and puerarin [6], have been reported to counteract the toxicity of 3-MCPD.

Dietary fiber is recognized as the seventh essential nutrient. IDF has been widely used as a dietary supplement in various food products. Increased consumption of IDF may be associated with the prevention of several conditions, including cancer, cardiovascular disease, inflammatory bowel disease, constipation, and type 2 diabetes mellitus [7]. IDF exhibits in vitro binding affinity for various heavy metals, including lead, mercury, cadmium, and arsenic, as well as for lard, cholesterol, and bile acids [8]. Furthermore, it has been shown to mitigate sodium dextran sulfate-induced enteritis in murine models [9], alleviate cadmium-induced toxicity in mice [10], relieve chronic constipation induced by loperamide [11], and reduce hepatic and renal injuries caused by nitrite exposure [12]. The benefits of IDF may be attributed to its bound polyphenolic antioxidants and adsorption capacity. Polyphenols in tea residue IDF mitigate aging induced by 3-MCPD [4]. Polyphenols can be cross-linked to cellulose and other components through covalent bonds, such as ester bonds formed between carboxyl groups and the hydroxyl groups, ether bonds with aromatic hydroxyl groups, or carbon–carbon (C–C) bonds [13]. These bound phenols are primarily released during intestinal digestion and are physiologically more beneficial than those released during gastric digestion [14]. Polyphenols exhibit a strong reactive oxygen species (ROS) scavenging effect. The toxicity mechanism of 3-MCPD may involve increasing ROS levels, leading to functional damage in cells or organs [15]. It has been suggested that both polyphenol-reduced IDF and IDF may play a similar mitigating role in dextran sulfate sodium toxicity by modulating the gut microbiota and intestinal barrier [9]. However, the impact of polyphenol-reduced IDF on 3-MCPD toxicity has not been reported.

Corn bran, a byproduct of corn processing, is rich in IDF but is often underutilized or discarded. This study aims to modify IDF extracted from corn bran using alkaline hydrogen peroxide (AHP) to remove phenolic groups, investigating the potential efficacy of polyphenol-reduced IDF in mitigating the toxicity of low-dose 3-MCPD.

## 2. Materials and Methods

### 2.1. Materials and Chemicals

3-MCPD (purity ≥ 98%) was purchased from Sigma-Aldrich Chemicals Co., Ltd. (Shanghai, China). A DAO kit was acquired from Shanghai Jianglai Biotechnology Co., Ltd. (Shanghai, China). The other materials are the same as those mentioned in previous literature [16]. Corn bran was sourced from Qiqihar Fufeng Biotechnology Co., Ltd. (Qiqihar, China). Enzymes including α-amylase (activity: 3.7 × 10^3^ U/g), glucoamylase (activity: 1.0 × 10^5^ U/g), and neutral protease (activity: 5.0 × 10^4^ U/g) were obtained from Beijing Solarbio Science & Technology Co., Ltd. (Beijing, China). Deionized water was used for the preparation of IDFs and all solutions, and 30% (*w*/*w*) hydrogen peroxide was also utilized. All chemicals used in this study were of analytical grade.

### 2.2. Preparation of IDF and IDF-AHP

The release process of IDF and IDF-AHP was based on a previous study [16]. Namely, 1 g corn bran powder was suspended in 30 mL deionized water with 0.8% neutral protease, incubated at 45 °C (pH 6.5) for 3 h, and then heated at 100 °C for 15 min to inactivate the enzyme. After cooling, 0.7% amylase–glucoamylase mixture (1:1 *w*/*w*) was added, followed by incubation at 45 °C (pH 5.5) for 100 min. After re-inactivation, the mixture was centrifuged (4000 rpm, 30 min), and the residue was washed (deionized water, 95% ethanol), dried (60 °C, 6 h), and sieved (80-mesh) to obtain native IDF. IDF-AHP (AHP-modified IDF) was prepared as followed: IDF was mixed with 1% H_2_O_2_ (1:20, g:mL), adjusted to pH 11.5 with 6 M NaOH, and stirred at 40 °C for 3 h. After centrifugation (5000 rpm, 20 min), the precipitate was washed and dried (60 °C) to yield IDF-AHP. Instruments used included a ZNCL-GS stirrer, TDL-5-A centrifuge, and DHG-9070 dryer (Zhengzhou, China). The composition of IDF and its water-holding properties are presented in Table 1.

### 2.3. FT-IR Spectroscopy

Fourier Transform Infrared (FT-IR) spectroscopy was employed to assess relative variations in chemical functional groups and molecular structures through the measurement of infrared radiation absorption or transmittance. First, 1 mg dry sample was mixed with 0.1 g KBr and pressed into a pellet under ambient conditions. The FT-IR spectrum was then recorded in the mid-infrared region, covering a wavenumber range of 4000–400 cm^−1^ [17].

### 2.4. Determination of Bound Phenol from IDF and IDF-AHP

The total phenol content was determined following previously reported methods [18] with minor modifications. A 50% ethanol solution was added at a ratio of 1:20 (g/mL), and the mixture was shaken in a water bath at 50 °C for 4 h. The ethanol extract was then centrifuged at 4000 rpm for 5 min. Then, 0.5 mL of Folin–Ciocalteu reagent was added to 0.5 mL of the ethanol extract of IDFs. After incubation in the dark at room temperature for 1 h, 0.4 mL of 10% (*m*/*v*) Na_2_CO_3_ solution was added, and the absorbance was measured at 765 nm. The results are expressed as milligrams of gallic acid equivalents per 100 g of dry weight (mg GAE/100 g DW).

### 2.5. Determination of Antioxidant Activity of IDF and IDF-AHP

The free radical scavenging activities of IDF and IDF-AHP were determined using the 2,2-diphenyl-1-picrylhydrazyl (DPPH) method, following a previously reported procedure with slight modifications [19]. Freshly prepared DPPH solution (0.1 mM, 2.9 mL) and the sample (ethanol extract of IDFs, 1.0 mL) were mixed and incubated for 30 min. After incubation, the mixture was centrifuged at 5000 rpm for 5 min, and the absorbance was measured at 517 nm. 

The DPPH radical scavenging activity was calculated using the following equation:

(1) $$x\;=\;\left(1-\frac{A_{i}-A_{j}}{A_{0}}\right)\;\times \;100\%$$

where *A_i_* is the absorbance of the sample and DPPH, *A_j_* is the absorbance of the sample and distilled water, and *A*_0_ is the absorbance of distilled water and DPPH.

### 2.6. Animals and Experimental Design

**Figure 1:**
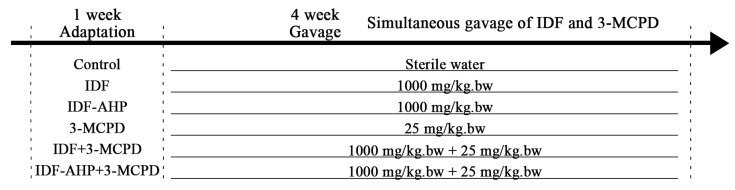
Experimental design.

The experimental protocol was approved by the Animal Ethics Committee of Qiqihar University (2024SP-004). Forty-eight 5-week-old female Kunming mice (Permission No. SCXK (liao) 2020-0001) were purchased from Liaoning Changsheng Biotechnology Co., Ltd. (Benxi, China). The mice were housed under a 12 h light/dark cycle at approximately 25 °C, with free access to food and water. The cages were placed in cage cabinets with doors, with three cages per level. Bedding consisted of autoclaved wood shavings, which were changed every two days. The food provided was a non-breeding type and was weighed both before and after the daily drop-off. They were randomly divided into six groups (*n* = 8 per group) based on weight, using the RAND function in Excel. The experimental design is shown in Figure 1: the control group received sterile water; the IDF and IDF-AHP groups each received 1000 mg/kg body weight (bw); the 3-MCPD group received 25 mg/kg bw; the IDF+3-MCPD group received both IDF (1000 mg/kg bw) and 3-MCPD (25 mg/kg bw); and the IDF-AHP+3-MCPD group received IDF-AHP (1000 mg/kg bw) and 3-MCPD (25 mg/kg bw). The 25 mg/kg bw dose is defined as a low dose based on previous studies [20]. In the current study, the lowest observed adverse effect level (LOAEL) was approximately 10 mg/kg bw in female mice, while the no observed adverse effect level (NOAEL) remained undetermined.

Each group of mice was administered 3-MCPDs and IDFs simultaneously using separate gavage needles. All follow-up animal testing was conducted using a single-blind method. After 28 days, the mice were sacrificed following the protocol of a previous study before sample collection [21]. All mice participating in the study were maintained in good health without disease, accidental injury, or death. Data points were not arbitrarily excluded for any measurements.

The human-equivalent dose for a 60 kg individual corresponding to 1000 mg/kg bw is 4.864 g/d, and for 25 mg/kg bw, it is 0.121 g/d. This amount (4.864 g/d) is less than the Chinese Nutritional Dietary Guidelines of 25–30 g/d.

A sufficient amount of feed was provided at a fixed time each day and weighed after 24 h. This procedure was repeated daily. Food intake is calculated using the following equation:

(2) $$m=\frac{\sum \left(m_{1}\left(Weight\;of\;feed\;input\right)-m_{2}\left(weight\;of\;feed\;after\;24\;h\right)\right)}{28}$$

### 2.7. Mouse Spontaneous Activity and Standing Activity

Mouse activity and standing activity were measured using a mouse activity monitor. The mice were placed in a cage with a grated lid, and spontaneous activity and standing activities were recorded for 5 min.

### 2.8. Open Field Test (OFT)

The open field test (OFT) employs a circular white arena measuring 45 cm in radius and 30 cm in height. Using the OFT-100 TM-Vision Behavioral Experiment System (Chengdu, China), each mouse was placed on one side of the apparatus and monitored for 5 min. After each trial, the arena was thoroughly cleaned with 75% ethanol to eliminate odor cues that might influence mouse behavior.

### 2.9. Accelerating Rotarod

Mice were placed on a rotating bar (ZB-200, Taimeng, Dongguan, China) rotating at 15 rpm for 5 min. The time at which each mouse fell off was recorded manually by the experimenter. Mice underwent three days of training before data collection to ensure they learned to walk on the rotating bar rather than cling to it or fall off. Alcohol wipes were used after testing to eliminate potential odor effects [22].

### 2.10. Colon Length, Histomorphological Scores, and Serum DAO Viability Assays

On the last day of the feeding cycle, the mouse colon was straightened and laid flat on measuring paper marked with a centimeter scale to measure its length, then photographed [9].

Colon tissues were fixed in 4% paraformaldehyde at 4 °C for 48 h, then paraffin-embedded and sectioned into 4 μm thick slices. The sections were stained with hematoxylin and eosin (H&E) solution of Solarbio (Beijing, China), examined under a Nikon microscope (Tokyo, Japan), and photographed. Histological scores of the colon were assigned based on the degree of inflammatory cell infiltration (Table 2) [23].

Serum DAO enzyme activity was quantified using a kit according to the manufacturer’s instructions.

### 2.11. 16S rDNA Sequencing Analysis of Gut Microbiota

Reference to the previous method with minor modifications [24]. From each group, 2 g of fresh mouse feces were collected from three randomly selected mice and stored in sterile 2 mL centrifuge tubes at −80 °C. Total DNA was extracted from the fecal samples using a Qiagen DNA extraction kit and then sent to Suzhou Panomix Biotechnology Co., Ltd. (Suzhou, China), for analysis of the mouse gut microbiota composition. The final DNA concentration and purity were quantified using a NanoDrop 2000 UV-Vis spectrophotometer (Thermo Scientific, Wilmington, DE, USA), and DNA quality was verified by 1% agarose gel electrophoresis. The V3-V4 hypervariable regions of the bacterial 16S rRNA gene were amplified with the primers 338F (5′-ACTCCTACGGGAGGCAGCAG-3′) and 806R (5′-GGACTACHVGGGTWTCTAAT-3′) by a thermocycler PCR system (GeneAmp 9700, ABI, Foster, CA, USA). 

PCR conditions: 95 °C denaturation (3 min); 27 cycles of 95 °C denaturation (30 s), 55 °C annealing (30 s), 72 °C elongation (45 s); 72 °C extension (10 min); final hold at 4 °C. Each 20 μL reaction contained 4 μL 5× TransStart FastPfu buffer, 2 μL 2.5 mM dNTPs, 0.8 μL each 5 μM primer, 0.4 μL TransStart FastPfu DNA Polymerase, and 10 ng template DNA, with reactions performed in triplicate. PCR products were gel-extracted from 2% agarose gels and purified using the AxyPrep DNA Gel Extraction Kit (Axygen Biosciences, Union City, CA, USA) according to the manufacturer’s instructions. Purified products were quantified using a Qubit 4 fluorometer (Thermo Fisher, Waltham, MA, USA). Equimolar amounts of purified amplicons were subjected to paired-end sequencing on an Illumina MiSeq PE300 platform (Illumina, San Diego, CA, USA) by Honsunbio Technology (Shanghai, China) following standard protocols.

Sequencing reads were demultiplexed (fastp v0.21.0: trim adaptors/quality score < Q20, discard <50 bp reads/ambiguous nucleotides) and merged (FLASH v1.2.7: ≥10 bp overlap, ≤20% overlap mismatch). Merged reads (retained for downstream analysis) were clustered into OTUs (97% similarity, UPARSE) after chimera removal. OTU taxonomy was assigned via RDP Classifier (reference database SILVA138, confidence ≥ 0.7), and rarefaction normalized OTU abundance across samples.

Demultiplexed reads were imported into QIIME2 (v2022.8), quality-filtered, denoised, and chimera-removed using DADA2. ASV taxonomy was assigned using the RDP Classifier against the reference database SILVA138 (confidence ≥ 0.7). Sample sequences were normalized to the lowest read count by random subsampling.

### 2.12. Statistical Analysis

All experimental data are presented as mean ± standard deviation (SD). Statistical analyses were performed using SPSS software 27. One-way analysis of variance (ANOVA) was conducted to evaluate differences among groups. Different letters denote statistically significant differences (*p* < 0.05).

General statistical analyses and result visualization were implemented using R software (version 4.1.3), with the assistance of multiple packages including vegan (v2.6-4), phyloseq (v1.38.0), tidyverse (v1.3.2), ggpubr (v0.5.0). Alpha diversity metrics, namely Chao1, Shannon, and Simpson indices, were calculated to evaluate community richness and evenness. To characterize beta diversity variations across groups, principal coordinates analysis (PCoA) was carried out based on Bray–Curtis dissimilarity matrices; permutational multivariate analysis of variance (PERMANOVA) was applied to determine the statistical significance of group differences. For inter-group comparisons of taxonomic relative abundances, the linear discriminant analysis (LDA) effect size (LEfSe) approach was employed, with the Kruskal–Wallis test set at *p* < 0.05 and the logarithmic LDA score threshold fixed at 2.0.

## 3. Results

### 3.1. FTIR Spectrum, Polyphenol Content, and Antioxidant Activity of IDF and IDF-AHP

**Figure 2:**
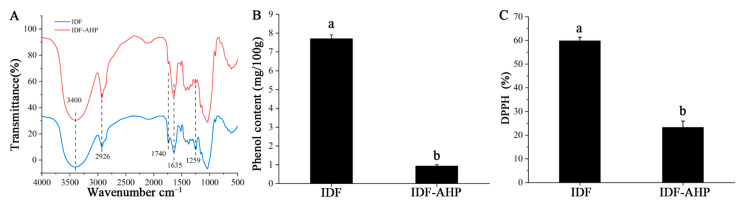
FT-IR spectrum, bound phenol content, and antioxidant activity of IDF and IDF-AHP. (**A**) FT-IR spectroscopy; (**B**) combined phenol content; (**C**) DPPH radical scavenging activity. Data are expressed as mean ± SD (*n* = 8). Different letters indicate significant differences (*p* < 0.05).

IDF and IDF-AHP exhibited similar characteristic peaks: cellulose O–H stretching at 3400 cm^−1^, sugar methylene C–H stretching at 2926 cm^−1^, and lignin benzene ring C=O stretching at 1635 cm^−1^ (Figure 2A), indicating that IDF and IDF-AHP retained polysaccharide features. The reduced intensity of the C=O bands at 1740 cm^−1^ (ester) and 1259 cm^−1^ (hydroxybenzene; Figure 2A) reflected the disruption of polyphenol–IDF ester bonds consumed during modification, suggesting a potential decrease in conjugated phenols [25]. IDF-AHP exhibited significantly lower levels of bound phenols than IDF (*p* < 0.05, Figure 2B), consistent with the effect of alkaline hydrogen peroxide treatment in reducing bound polyphenols [26]. Combined phenols have been shown to possess strong antioxidant activity [14]. Correspondingly, IDF-AHP exhibited a significant decrease in DPPH radical scavenging activity, from 59.88% to 23.33%, compared to IDF (*p* < 0.05, Figure 2C). This reduction likely results from the depletion of polyphenols under alkaline conditions and the oxidation of reducing groups by hydrogen peroxide, thereby decreasing radical inhibition.

### 3.2. Body Weight and Feed Intake

**Figure 3:**
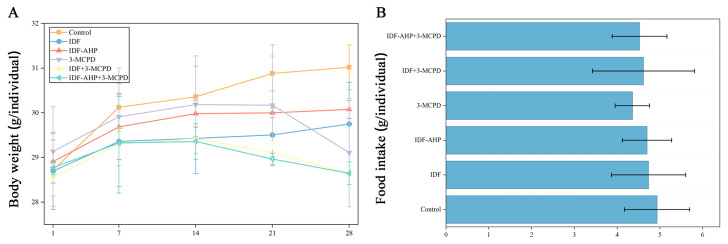
Animal experiment schedule, body weight, and food intake in the six groups. (**A**) body weight; (**B**) food intake. Data are expressed as mean ± SD (*n* = 8).

The effects of different gavage strategies on body weight are presented in Figure 3A. Body weights in the control, IDF, and IDF-AHP groups showed a consistent upward trend. In contrast, the 3-MCPD, IDF+3-MCPD, and IDF-AHP+3-MCPD groups exhibited increased body weight until Day 14, followed by a decline thereafter. Body weights on day 28 were ranked in descending order as follows: control group > IDF group > IDF-AHP group > 3-MCPD group > IDF+3-MCPD group > IDF-AHP+3-MCPD group (*p* > 0.05). This observation may be attributed to the laxative effect of IDF, as IDF, IDF-AHP, IDF+3-MCPD, and IDF-AHP+3-MCPD increased fecal moisture content in mice, which may be related to the increased fecal moisture content observed in preliminary measurements. Previous studies have indicated that the toxicity-alleviating effect of IDFs may be associated with shortened defecation time, increased fecal moisture, and enhanced intestinal motility [27]. Additionally, the observed changes in body weight were not due to alterations in food intake, as no significant differences in food consumption were noted throughout the feeding period (*p* > 0.05) (Figure 3B).

### 3.3. IDFs Enhance the Motor Activity of Mice Exposed to Low-Dose 3-MCPD

**Figure 4:**
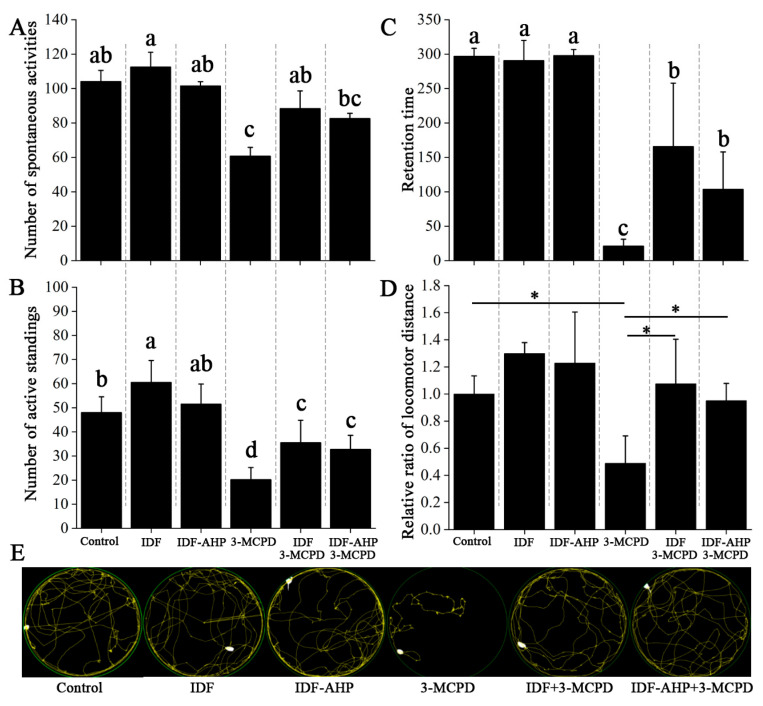
Alleviating effects of IDF and IDF-AHP on 3-MCPD-induced motor impairment. (**A**) Number of spontaneous activities; (**B**) number of active standings; (**C**) retention time; (**D**) locomotor distance; (**E**) movement trajectory diagram. Data are expressed as mean ± SD (*n* = 8). Different letters or * indicate significant differences (*p* < 0.05).

Previous studies have demonstrated that 3-MCPD induces paralysis and impairs motor activity [28,29]. Mouse mobility was assessed using rotarod, spontaneous activity, and open field tests (Figure 4). Mice in the IDF and IDF-AHP groups showed no significant differences in spontaneous activity, active standing, or retention time compared to the control group (*p* > 0.05, Figure 4A–C). Compared to the 3-MCPD group, the IDF+3-MCPD group exhibited significantly increased spontaneous activity, active standing, and retention time (*p* < 0.05, Figure 4A,B). The IDF-AHP+3-MCPD group also showed improvements in active standing and retention time (*p* < 0.05, Figure 4A–C). The open field test (Figure 4D,E) revealed no significant difference in locomotor distance between the IDF, IDF-AHP, and control groups (*p* > 0.05). However, the IDF+3-MCPD and IDF-AHP+3-MCPD groups exhibited significantly increased locomotor distances compared to the 3-MCPD group (*p* < 0.05). These findings indicate that IDF or IDF-AHP mitigates the toxicity of low-dose 3-MCPD and partially restores locomotor function.

The mechanism by which IDFs alleviate low-dose 3-MCPD-induced motor impairment remains unclear. Some studies suggest that ROS play a key role [30,31]. Polyphenols are effective in reducing ROS levels. However, IDF-AHP, which has lower antioxidant activity and phenolic content, alleviates low-dose 3-MCPD-induced motor impairment. This mitigation of 3-MCPD toxicity may not occur through ROS pathways; instead, its mechanism may involve protecting the intestinal barrier [32,33] and modulating gut microbiota [32]. Notably, 3-MCPD-induced dyskinesia may not be related to starvation-induced weakness, as food intake was not significantly affected (Figure 3B).

### 3.4. IDFs Improve the Intestinal Barrier

**Figure 5:**
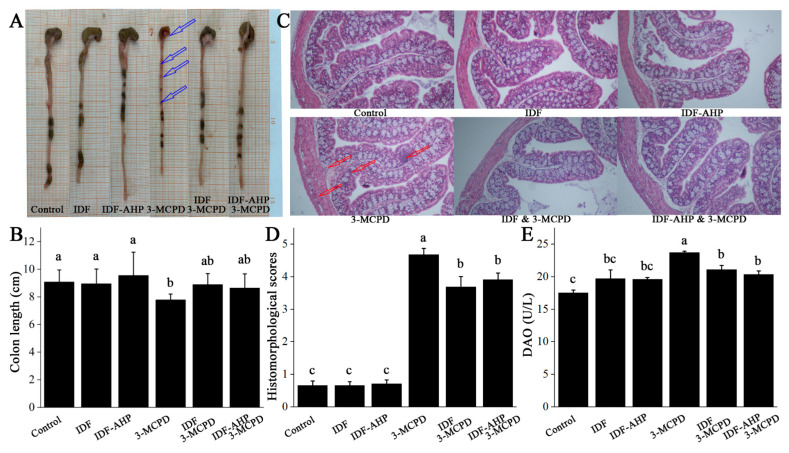
Effects of IDF and IDF-AHP on intestinal barrier in 3-MCPD-induced mice. (**A**) Representative photographs; (**B**) colon length; (**C**) HE staining; (**D**) histochemical scores; (**E**) DAO content. Data are expressed as mean ± SD (*n* = 8). Hollow blue arrows, obvious blood; hollow red arrows, inflammatory cell infiltration. Different letters represent significant differences (*p* < 0.05).

Representative images and colon length data are shown in Figure 5A,B. The 3-MCPD group exhibited significantly shorter colons than the control group (*p* < 0.05), accompanied by obvious blood, a slender morphology (indicated by hollow blue arrows), and transparent intestinal walls. Compared to the 3-MCPD group, colon lengths in the IDF+3-MCPD and IDF-AHP+3-MCPD groups were slightly increased (*p* > 0.05). HE staining revealed inflammatory cell infiltration in the mucosal, submucosal, and muscular layers of colons from the 3-MCPD group (indicated by hollow red arrows, Figure 5C), with infiltration scores significantly higher than those of the control group (*p* < 0.05, Figure 5D). In the IDF+3-MCPD and IDF-AHP+3-MCPD groups, inflammatory cell infiltration was significantly reduced, with scores significantly lower than those of the 3-MCPD group (*p* < 0.05). DAO levels in the 3-MCPD group were significantly higher than those in the control group (*p* < 0.05). In contrast, serum DAO levels were significantly reduced in the IDF+3-MCPD and IDF-AHP+3-MCPD groups (*p* < 0.05, Figure 5E).

It has been shown that 3-MCPD induces inflammatory cell infiltration in the renal mesenchyme [34]. In the present study, 3-MCPD triggers inflammatory cell infiltration in the colon. This inflammation leads to increased intestinal permeability [35], which prompts intestinal epithelial cells to release DAO, a marker of intestinal barrier damage [36]. Consequently, serum DAO levels rise. Furthermore, it has been demonstrated that reducing inflammatory cell infiltration reduces DAO levels [32], confirming the association between the two and intestinal barrier damage. The addition of IDF and IDF-AHP significantly reduced 3-MCPD-induced inflammatory cell infiltration in the muscular and submucosal layers of the colon, as well as serum DAO levels. In conclusion, IDF and IDF-AHP may be associated with improved intestinal barrier function by reducing colonic inflammation and mucosal damage [37].

### 3.5. IDFs Modulate the Gut Microbial Community

**Figure 6:**
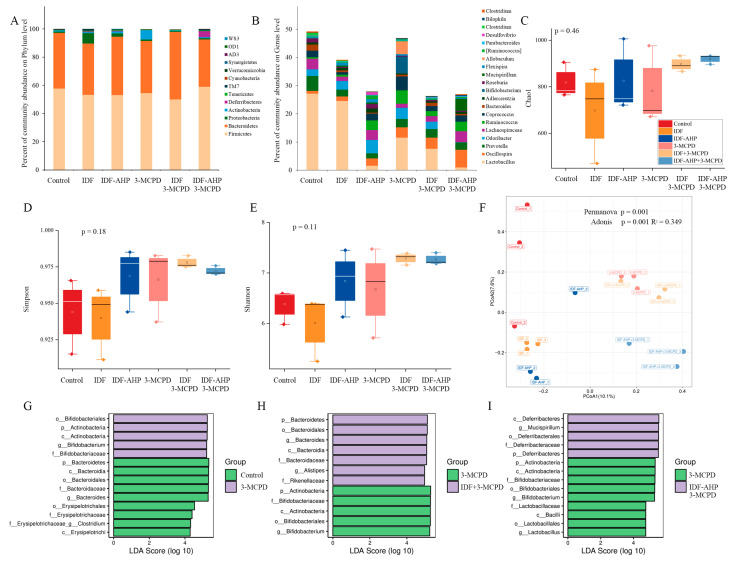
Analysis result of 16s RNA among the six groups. (**A**,**B**) Microbial composition at phylum and genus level. (**C**–**E**) Box plot of the α-diversity index of the gut microbiota. (**F**) PCoA plot. (**G**–**I**) LEfSe analysis of the gut microbiota. Data are expressed as mean or mean ± SD (*n* = 3).

The obtained operational taxonomic units (OTUs) were classified into 13 bacterial phyla, and the distribution of the phyla with the highest relative abundance across the six groups is shown in Figure 6A. The most abundant phylum in all six groups (control, IDF, IDF-AHP, 3-MCPD, IDF+3-MCPD, and IDF-AHP+3-MCPD) was *Firmicutes*, with relative abundances of 57.78%, 53.27%, 52.99%, 54.46%, 50.06%, and 58.91%, respectively. Among the four groups (control, IDF, 3-MCPD, and IDF+3-MCPD), the most abundant genus was *Lactobacillus*; in the IDF-AHP group, it was *Odoribacter*; and in the IDF-AHP+3-MCPD group, *Oscillospira* was predominant (Figure 6B).

The Chao1, Simpson, and Shannon indices are presented in Figure 6C–E, respectively. The Chao1 index reflects species richness, while the Simpson and Shannon indices measure community diversity [38]. The *p*-values for the Chao1, Simpson, and Shannon indices were 0.48, 0.16, and 0.11, respectively, indicating that neither IDF nor IDF-AHP significantly affected gut microbial α-diversity. The overall abundance and structural diversity of gut microorganisms did not differ significantly among the groups.

β-diversity is an macro-metric marker used to assess differences in microbial communities between samples [39]. Significant differences in microbial composition were observed among the groups (*p* = 0.001, Adonis, R^2^ = 0.349). In the PCoA plot, the IDF+3-MCPD group showed slight separation from the 3-MCPD group, whereas the IDF-AHP group exhibited distinct separation from the 3-MCPD group (Figure 6F).

Multilevel linear discriminant analysis effect size (LEfSe) was used to identify gut microbes that differed significantly among the six groups. The threshold for LDA scores was set at ≥2, with a significance level of *p* < 0.05. The results of the LEfSe analyses are shown in Figure 6G–I. LEfSe analysis revealed significant differences between the control and 3-MCPD groups in the phyla *Bacteroidetes*, *Firmicutes*, and *Actinobacteria*. *Clostridium* and *Bacteroides* were the dominant genera in the control group, whereas *Bifidobacterium* predominated in the 3-MCPD group (Figure 6G). Comparisons between the IDF+3-MCPD and 3-MCPD groups showed significant differences in *Bacteroidetes* and *Actinobacteria*; *Alistipes* and *Bacteroides* were dominant in the IDF+3-MCPD group, while *Bifidobacterium* remained dominant in the 3-MCPD group (Figure 6H). Additionally, the IDF-AHP+3-MCPD group differed significantly from the 3-MCPD group in *Deferribacteres*, *Firmicutes*, and *Actinobacteria*, with *Mucispirillum* as the dominant genus in the IDF-AHP+3-MCPD group, and *Lactobacillus* and *Bifidobacterium* as dominant genera in the 3-MCPD group (Figure 6I). Studies have demonstrated that *Bifidobacterium* is associated with the alleviation of nerve damage [40]. Therefore, *Bifidobacterium* differed significantly between the 3-MCPD group and the control group, the IDF+3-MCPD group, and the IDF-AHP+3-MCPD group and was the dominant genus in the 3-MCPD group. In the IDF+3-MCPD group, *Alistipes* and *Bacteroides* emerged as dominant genera (replacing *Bifidobacterium*) and may be relevant to the mitigation of 3-MCPD toxicity. Studies have demonstrated that *Alistipes* and *Bacteroides* are associated with the alleviation of motor deficits [41]. Compared to the 3-MCPD group, *Mucispirillum* emerged as the dominant genus in the IDF-AHP+3-MCPD group. The increased abundance of *Mucispirillum* may be associated with neurotransmitter restoration [42]. In summary, both IDF and IDF-AHP modulated the gut microbial community composition in mice exposed to 3-MCPD.

## 4. Discussion

Although IDF and polyphenol-reduced IDF exhibited similar mitigation of 3-MCPD toxicity, this does not imply that the polyphenols in IDF are dispensable. The effects of polyphenols and IDF may be synergistic [43]. IDF with reduced polyphenols showed a significant and substantial decrease in functions such as antioxidant activity. On one hand, there is still residual polyphenol that may play a role, and on the other, IDF itself may produce antioxidant components to counteract the toxicity of 3-MCPD during intestinal fermentation [44].

The brain and nerves are among the target organs affected by 3-MCPD [20]. Protective effects of intestinal flora and short-chain fatty acids, regulated by probiotics and complex dietary fiber, on the brain and nervous system have been reported [45]. Follow-up experiments examining brain tissue structure, apoptosis-related genes, and oxidative stress markers in 3-MCPD-exposed brains, combined with data on gut flora and short-chain fatty acids, may provide direct evidence elucidating the mechanisms underlying the toxicity-mitigating effects of IDF and IDF-AHP on 3-MCPD.

3-MCPD is a common contaminant found in processed foods such as soy sauce, refined oils, and heat-treated grains. Human exposure to 3-MCPD at a dose of 25 mg/kg body weight is unlikely due to detection limits. However, it is possible for humans to consume up to 10 g of dietary fiber per day. Additionally, humans metabolize 3-MCPD into 3-chlorolactic acid [46], which may be metabolized more rapidly than in mice. Therefore, it is advisable to increase the intake of IDF while remaining within the established detection limits.

Industrial IDF extraction and AHP modification processes may consume large amounts of water, acids, and bases, and managing the resulting wastewater presents a significant challenge. In our previous study [16], we confirmed that the modified IDF contains no hydrogen peroxide residue; however, long-term studies are needed to monitor residue levels and assess biosafety. These further investigations will support the extraction of high-fiber components from cereal by-products, aligning with the principles of a circular economy and waste utilization.

## 5. Conclusions

In this study, we compared native corn bran IDF and alkaline hydrogen peroxide-modified, polyphenol-depleted IDF (IDF-AHP) in a mouse model of 3-MCPD-induced toxicity. Despite having reduced phenolic content and antioxidant activity, IDF-AHP still alleviated motor impairment and intestinal damage, highlighting roles for the intestinal barrier and gut microbiota beyond direct ROS scavenging. Polyphenol-reduced IDF exhibited lower phenolic content and antioxidant properties and alleviated low-dose 3-MCPD-induced motor impairment by improving intestinal barrier function and intestinal flora composition. Relative to IDF, it also exhibited similar effects and outperformed IDF-AHP in some indices. These results not only suggest that the ROS elimination pathway can mitigate the toxicity induced by low-dose 3-MCPD, but also that the intestinal barrier and intestinal flora structure may be important factors in 3-MCPD toxicity. The differential bacteria *Alistipes*, *Bacteroides* and *Mucispirillum* may be associated with recovery from motor impairments induced by 3-MCPD.

Although these results suggest a protective effect of IDF and polyphenol-reduced IDF, certain limitations must be acknowledged. The experiment was conducted using a low dose of 3-MCPD and was not validated at higher doses. Additionally, DPPH, as an in vitro antioxidant assay, may not fully represent biological antioxidant indicators such as ROS and antioxidant enzymes. Dietary MCPD is typically ingested in an esterified form, although it is metabolized in vivo to 3-MCPD. Similar validation in other species is also necessary. Microbiota analysis was conducted on three animals per group (*n* = 3), which may have limited the ability to detect changes in diversity and relative abundance.

Future studies should focus on the patterns of change in the brain–gut axis.

## Figures and Tables

**Table 1:** IDF and IDF-AHP basic components.

Form	IDF	IDF-AHP
Crude protein (%)	3.05 ± 0.04	1.46 ± 0.00
Moisture (g/100g)	8.09 ± 0.05	8.85 ± 0.05
Starch (g/100g)	0.92 ± 0.02	1.04 ± 0.07
Ash (g/100g)	1.08 ± 0.01	2.16 ± 0.01
WHC	3.90 ± 0.02	9.20 ± 0.08

**Table 2:** Colon histological score standard.

Inflammatory Cell Infiltration	Score
Mucosa, normal	0
Mucosa, mild	1
Mucosa, modest	2
Mucosa, severe	3
Submucosa, normal	0
Submucosa, mild to modest	1
Submucosa, severe	2
Muscularis, normal	0
Muscularis, modest to severe	1
Epithelial damage	
NO change	0
Epithelial infiltration	1
Crypt abscesses, erosion	2
Ulcerations	3
Mucosal architecture/atypia	
NO change	0
Simple hyperplasia	1
Low- and high grade dysplasia	2
Invasive carcinoma	3
Max. Score	12

## Data Availability

The original contributions presented in the study are included in the article, further inquiries can be directed to the corresponding author.

## Abbreviations

IDF	in insoluble dietary fiber
IDF-AHP	polyphenol-reduced IDF; IDF modified with alkaline hydrogen peroxide
3-MCPD	3-chloro-1,2-propanediol
DAO	diamine oxidase
DPPH	2,2-diphenyl-1-picrylhydrazyl
ROS	reactive oxygen species
bw	body weight
OTUs	operational taxonomic units

## References

[1] Ahmadpourmir H., Velayati M., Tsitsimpikou C., Tsatsakis A., Tzatzarakis M., Sahranavard T., Taghizadeh S.F., Rezaee R. (2024). 3-Monochloropropane-1,2-Diol (3-MCPD) (Free and Esterified) in Edible Oil, Soy Sauce and Infant Formula: A Systematic Review of the Occurrence and Employed Analytical Approaches. Microchem. J..

[2] Fattore E., Lanno A., Danieli A., Stefano S., Passoni A., Roncaglioni A., Bagnati R., Davoli E. (2023). Toxicology of 3-Monochloropropane-1,2-Diol and Its Esters: A Narrative Review. Arch. Toxicol..

[3] Arris F.A., Thai V.T.S., Manan W.N., Sajab M.S. (2020). A Revisit to the Formation and Mitigation of 3-Chloropropane-1,2-Diol in Palm Oil Production. Foods.

[4] Liu D., Song Y., Chen X., Xie J., Chen Y., Hu X., Xie J., Yu Q. (2024). Multi-Omics Reveals the Anti-Aging Effects and Mechanisms of Bound Polyphenols from Tea Residue Dietary Fiber in 3-MCPD-Induced *Caenorhabditis elegans*. Food Biosci..

[5] Jiang X., Zhu C., Li X., Sun J., Tian L., Bai W. (2018). Cyanidin-3-O-Glucoside at Low Doses Protected against 3-Chloro-1,2-Propanediol Induced Testis Injury and Improved Spermatogenesis in Male Rats. J. Agric. Food Chem..

[6] Wang R., Tao M., Zhu Y., Fan D., Wang M., Zhao Y. (2021). Puerarin Inhibited 3-Chloropropane-1,2-Diol Fatty Acid Esters Formation by Reacting with Glycidol and Glycidyl Esters. Food Chem..

[7] Tian M., Pak S., Ma C., Ma L., Rengasamy K.R.R., Xiao J., Hu X., Li D., Chen F. (2024). Chemical Features and Biological Functions of Water-Insoluble Dietary Fiber in Plant-Based Foods. Crit. Rev. Food Sci. Nutr..

[8] Zhang N., Huang C., Ou S. (2011). In Vitro Binding Capacities of Three Dietary Fibers and Their Mixture for Four Toxic Elements, Cholesterol, and Bile Acid. J. Hazard. Mater..

[9] Yin W., Liu M., Jin Z., Hao Z., Liu C., Liu J., Liu H., Zheng M., Cai D. (2025). Ameliorative Effects of Insoluble Dietary Fiber and Its Bound Polyphenols from Adzuki Bean Seed Coat on Acute Murine Colitis Induced by DSS: The Inflammatory Response, Intestinal Barrier and Gut Microbiota. Int. J. Biol. Macromol..

[10] Li X., Ren M., Zhang X., Wang L. (2022). Insoluble Dietary Fiber (Non-Starch Polysaccharides) from Rice Bran Attenuates Cadmium-Induced Toxicity in Mice by Modulating the Gut Microbiota and Alleviating Liver and Kidney Injury. Food Biosci..

[11] Reis V.H.d.O.T., de Melo V.X., da Silva M.L.R., Filho P.S.L., Portugal L.C., Sartoratto A., Rafacho B.P.M., Cazarin C.B.B., Cordeiro L.M.C., dos Santos E.F. (2025). Insoluble Dietary Fibers from *Hancornia speciosa* Alleviates Chronic Constipation on Experimental Loperamide-Induced Model. Int. J. Biol. Macromol..

[12] Zhang R., Wang J., Wang Y., Jing W., Lyu B., Wang S., Li J., Yang H., Yu H. (2025). Hydrochloric Acid-Modified High-Purity Soybean Insoluble Dietary Fiber from Okara Attenuates NO_2_− Induced Hepatic and Renal Injury in Mice. J. Food Sci..

[13] Fernandes A., Mateus N., de Freitas V., Fernandes A., Mateus N., Freitas V. (2023). Polyphenol-Dietary Fiber Conjugates from Fruits and Vegetables: Nature and Biological Fate in a Food and Nutrition Perspective. Foods.

[14] Liu M., Liu X., Luo J., Bai T., Chen H. (2021). Effect of Digestion on Bound Phenolic Content, Antioxidant Activity and Hypoglycemic Ability of Insoluble Dietary Fibre from Four Triticeae Crops. J. Food Biochem..

[15] Zhang R., Guan S., Meng Z., Deng X., Lu J. (2024). 3-MCPD Induces Renal Cell Pyroptosis and Inflammation by Inhibiting ESCRT-III-Mediated Cell Repair and Mitophagy. J. Agric. Food Chem..

[16] Song Z., Dai H., Bo L., Song C., Liu X., Ren J. (2024). Set Yogurt Incorporated with Insoluble Dietary Fiber Maintains Non-Sedimentation: Combined Alkaline Hydrogen Peroxide Modification and High-Pressure Homogenization Process. LWT.

[17] Luo M., Wang C., Wang C., Xie C., Hang F., Li K., Shi C. (2022). Effect of Alkaline Hydrogen Peroxide Assisted with Two Modification Methods on the Physicochemical, Structural and Functional Properties of Bagasse Insoluble Dietary Fiber. Front. Nutr..

[18] Li Y., Chen X., Wang G., Xu L., Liu Y., Yuan C., Li J. (2025). The Release Patterns and Potential Prebiotic Characteristics of Soluble and Insoluble Dietary Fiber-Bound Polyphenols from Pinot Noir Grape Pomace In Vitro Digestion and Fermentation. Food Chem. X.

[19] Si J., Xie J., Zheng B., Xie J., Chen Y., Yang C., Sun N., Wang Y., Hu X., Yu Q. (2023). Release Characteristic of Bound Polyphenols from Tea Residues Insoluble Dietary Fiber by Mixed Solid-State Fermentation with Cellulose Degrading Strains CZ-6 and CZ-7. Food Res. Int..

[20] Lee B.-S., Park S.-J., Kim Y.-B., Han J.-S., Jeong E.-J., Moon K.-S., Son H.-Y. (2015). A 28-Day Oral Gavage Toxicity Study of 3-Monochloropropane-1,2-Diol (3-MCPD) in CB6F1-Non-Tg rasH2 Mice. Food Chem. Toxicol..

[21] Gan Q., Shao J., Sun T. (2025). The Inhibitory Effects of Anti-GPC3 Antibody on Wnt/β-Catenin Signaling Pathway as a Biological Therapy in Liver Cancer. Mol. Biotechnol..

[22] Deacon R.M.J. (2013). Measuring Motor Coordination in Mice. J. Vis. Exp. (JoVE).

[23] Remke M., Groll T., Metzler T., Urbauer E., Kövilein J., Schnalzger T., Ruland J., Haller D., Steiger K. (2024). Histomorphological Scoring of Murine Colitis Models: A Practical Guide for the Evaluation of Colitis and Colitis-Associated Cancer. Exp. Mol. Pathol..

[24] Feng X., Zhang Y., Feng J., Li Z., Zhang Z., Zhu L., Zhou R., Wang H., Dai X., Liu Y. (2025). Exploring changes in metabolites and fecal microbiota of advanced gastric cancer based on plasma metabolomics and 16S rDNA sequencing. Heliyon.

[25] Liu S., Jia M., Chen J., Wan H., Dong R., Nie S., Xie M., Yu Q. (2019). Removal of Bound Polyphenols and Its Effect on Antioxidant and Prebiotics Properties of Carrot Dietary Fiber. Food Hydrocoll..

[26] Gao Q., Zhou X., Ma R., Lin H., Wu J., Peng X., Tanokura M., Xue Y. (2021). Hydrogen Peroxide Modification Affects the Structure and Physicochemical Properties of Dietary Fibers from White Turnip (*Brassica rapa* L.). Sci. Rep..

[27] Yang W., Gao X., Lin J., Liu L., Peng L., Sheng J., Xu K., Tian Y. (2024). Water-Insoluble Dietary Fiber from Walnut Relieves Constipation through *Limosilactobacillus Reuteri*-Mediated Serotonergic Synapse and Neuroactive Ligand-Receptor Pathways. Int. J. Biol. Macromol..

[28] Ford W.C.L., Waites G.M.H. (1982). Activities of Various 6-Chloro-6-Deoxysugars and (S)α-Chlorohydrin in Producing Spermatocoeles in Rats and Paralysis in Mice and in Inhibiting Glucose Metabolism in Bull Spermatozoa in Vitro. Reproduction.

[29] Liu D., Song Y., Zheng B., Xie J., Chen Y., Xie J., Chen X., Yu Q. (2024). EGCG Alleviates the Aging Toxicity Induced by 3-MCPD via IIS Pathway in Caenorhabditis Elegans with Abnormal Reproduction and Heat Shock Protein. J. Agric. Food Chem..

[30] Cai Y., Liu Z., Gao T., Hu G., Yin W., Wāng Y., Zhao L., Xu D., Wang H., Wei T. (2023). Newly Discovered Developmental and Ovarian Toxicity of 3-Monochloro-1,2-Propanediol in *Drosophila Melanogaster* and Cyanidin-3-*O*-Glucoside’s Protective Effect. Sci. Total Environ..

[31] Zhang R., Guan S., Meng Z., Zhang D., Lu J. (2024). Ginsenoside Rb1 Alleviates 3-MCPD-Induced Renal Cell Pyroptosis by Activating Mitophagy. Food Chem. Toxicol..

[32] Chen M., Huang H., Zhou P., Zhang J., Dai Y., Yang D., Fan X., Pan H. (2019). Oral Phosphatidylcholine Improves Intestinal Barrier Function in Drug-Induced Liver Injury in Rats. Gastroenterol. Res. Pract..

[33] Liu Z., He Y., Wang Y., Ren K., Xia P., Xie B., Wei T. (2025). Oxidative Stress Caused by 3-Monochloro-1,2-Propanediol Provokes Intestinal Stem Cell Hyperproliferation and the Protective Role of Quercetin. Ecotoxicol. Environ. Saf..

[34] Mahmoud Y.I., Abo-Zied F.S., Salem S.T. (2019). Effects of Subacute 3-Monochloropropane-1,2-Diol Treatment on the Kidney of Male Albino Rats. Biotech. Histochem..

[35] Van Houten J., Wessells R., Lujian H., DiCarlo S. (2015). My gut feeling says rest: Increased intestinal permeability contributes to chronic diseases in high-intensity exercisers. Med. Hypotheses.

[36] Miyoshi J., Miyamoto H., Goji T., Taniguchi T., Tomonari T., Sogabe M., Kimura T., Kitamura S., Okamoto K., Fujino Y. (2015). Serum Diamine Oxidase Activity as a Predictor of Gastrointestinal Toxicity and Malnutrition Due to Anticancer Drugs. J. Gastroenterol. Hepatol..

[37] Zhang R., Duan S., Ma X., Guo Z., Szeto I.M.-Y., Shi X., Zhao C., Yan Y., Li B. (2025). Synergistic Effect of 2’-Fucosyllactose and Osteopontin on Intestinal Mucosal Immunity Injury. Food Funct..

[38] Liang Y., Yu W., Wang H., Yao L., He Z., Sun M., Feng T., Yu C., Yue H. (2024). Flash Extraction of Ulvan Polysaccharides from Marine Green Macroalga *Ulva Linza* and Evaluation of Its Antioxidant and Gut Microbiota Modulation Activities. Int. J. Biol. Macromol..

[39] Lozupone C., Lladser M.E., Knights D., Stombaugh J., Knight R. (2011). UniFrac: An Effective Distance Metric for Microbial Community Comparison. ISME J..

[40] Reiriz M., Beltrán-Velasco A.I., Echeverry-Alzate V., Martínez-Miguel E., Gómez-Senent S., Uceda S., Clemente-Suárez V.J. (2025). Bifidobacterium Infantis and Bifidobacterium Breve Improve Symptomatology and Neuronal Damage in Neurodegenerative Disease: A Systematic Review. Nutrients.

[41] Lee Y.Z., Cheng S.-H., Chang M.-Y., Lin Y.-F., Wu C.-C., Tsai Y.-C. (2023). Neuroprotective Effects of Lactobacillus Plantarum PS128 in a Mouse Model of Parkinson’s Disease: The Role of Gut Microbiota and MicroRNAs. Int. J. Mol. Sci..

[42] Mo L., Jing H., Du X., Zhao C., Lin Y., Li J., Wang H. (2023). Goat and Cow Milk Differ in Altering the Microbiota Composition and Neurotransmitter Levels in Insomnia Mouse Models. Food Funct..

[43] Zheng B., Zhao X., Ao T., Chen Y., Xie J., Gao X., Liu L., Hu X., Yu Q. (2024). The Role of Bound Polyphenols in the Anti-Obesity Effects of Defatted Rice Bran Insoluble Dietary Fiber: An Insight from Multi-Omics. Food Chem..

[44] Ribeiro T.B., Costa C.M., Bonifacio-Lopes T., Silva S., Veiga M., Monforte A.R., Nunes J., Vicente A.A., Pintado M. (2021). Prebiotic Effects of Olive Pomace Powders in the Gut: In Vitro Evaluation of the Inhibition of Adhesion of Pathogens, Prebiotic and Antioxidant Effects. Food Hydrocoll..

[45] Ren M., Li H., Fu Z., Li Q. (2022). Centenarian-Sourced Lactobacillus Casei Combined with Dietary Fiber Complex Ameliorates Brain and Gut Function in Aged Mice. Nutrients.

[46] Bergau N., Zhao Z., Abraham K., Monien B.H. (2021). Metabolites of 2- and 3-Monochloropropanediol (2- and 3-MCPD) in Humans: Urinary Excretion of 2-Chlorohydracrylic Acid and 3-Chlorolactic Acid after Controlled Exposure to a Single High Dose of Fatty Acid Esters of 2- and 3-MCPD. Mol. Nutr. Food Res..

